# Supplementary figures and images for: The acceptability and uptake of smartphone tracking for COVID-19 in Australia
Source: PLoS One. 2021 Jan 22;16(1):e0244827. doi: 10.1371/journal.pone.0244827 (PMC7822556; doi:10.1371/journal.pone.0244827)

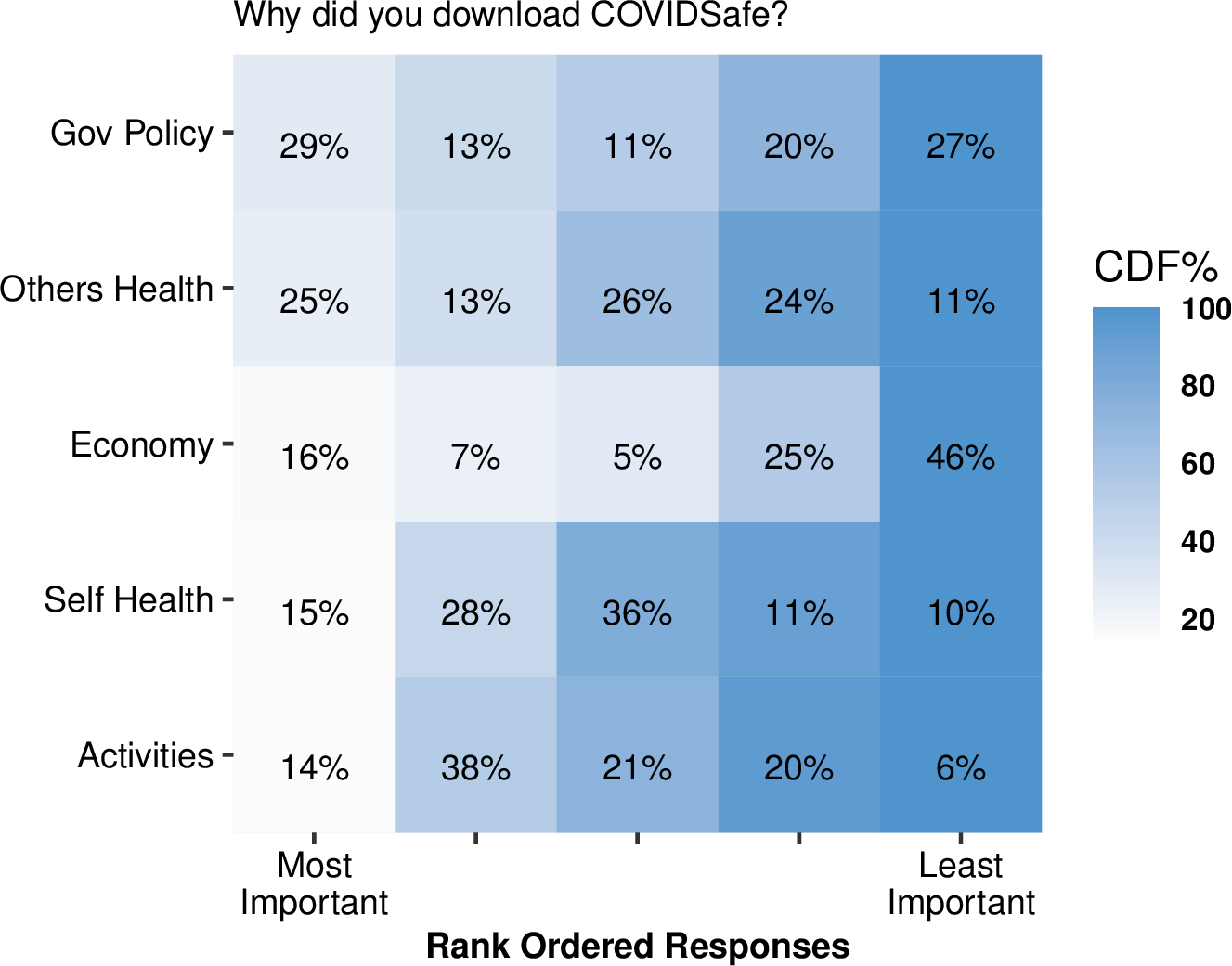

Supplement: S1 Fig — Participants were asked to rank order from most-important to least-important the five reasons presented on the y-axis of S1 Fig. The cumulative responses probabilities for each item are displayed in blue and the percentage of responses at each ranked-position are displayed by text in each cell, for example, in the first ranked-position Government policy was viewed as most important (29%) followed by others health (25%). Returning to normal activities was most frequently ranked in the second position (38%) followed by self health (28%). To show which items were viewed as most important overall, the cumulative percentage of responses to each item are shaded in blue, with cumulative percentages increasing from left-to-right (lighter to darker). For example, return to normal activities, self health, and others health were perceived as the important reasons overall, accruing the most responses in the first three rank-positions. Overall, the economy was ranked the least-important reason for down-loading COVIDSafe. (TIF) [file pone.0244827.s001.tif]

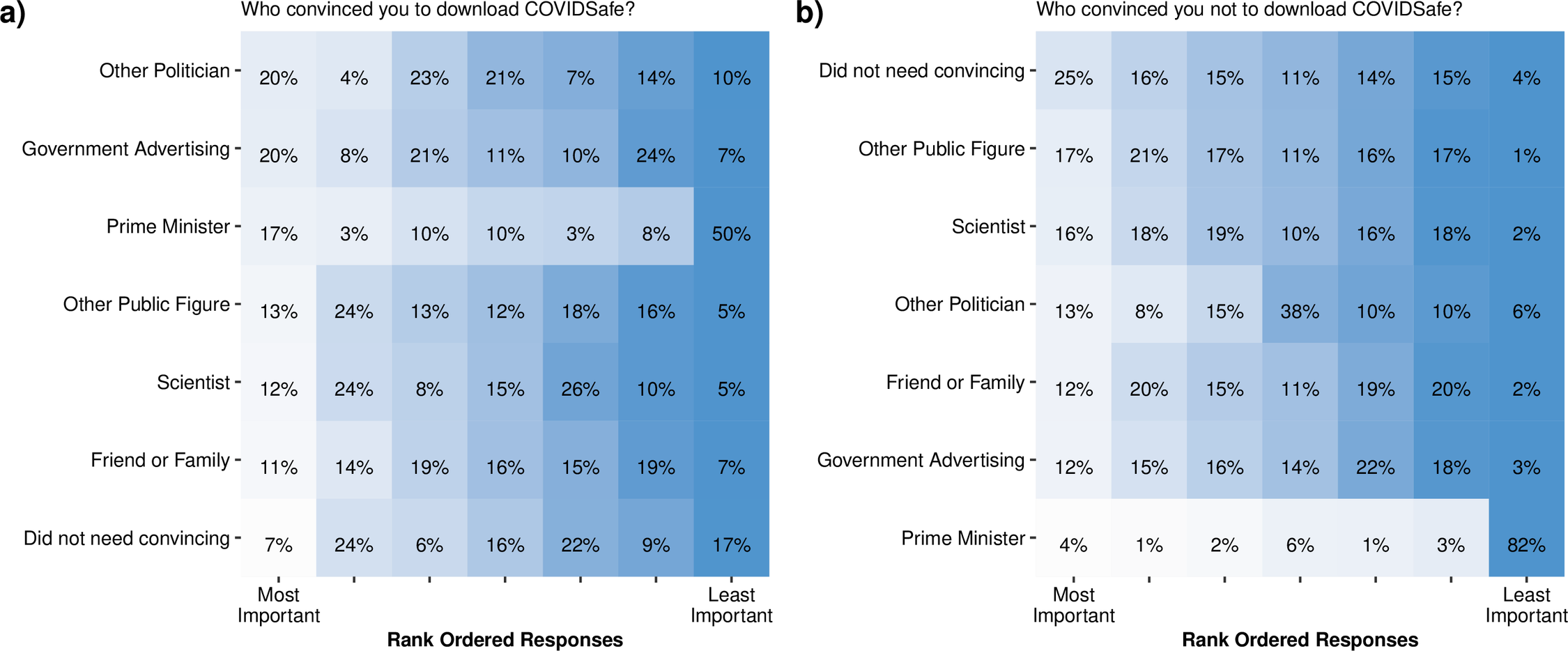

Supplement: S2 Fig — Each plot displays data collapsed across Samples 3 and 4; response percentages are displayed in text and the cumulative responses probabilities for each item are displayed in blue. When asked, most participants responded that other politicians, government advertising and the Prime Minister were the primary individuals who convinced them to download COVID Safe (S2a Fig). Public figures, scientists and did not need convincing were frequently ranked in the first three-positions. The Prime Minister was ranked as least important by 50% of respondents. Most participants who did not download COVIDSafe indicated they ‘did not need convincing’ or were convinced by other public figures and scientists (S2b Fig). Friends and family were frequently ranked in the top-three positions. The Prime Minister was ranked least important by 82% of respondents. (TIF) [file pone.0244827.s002.tif]

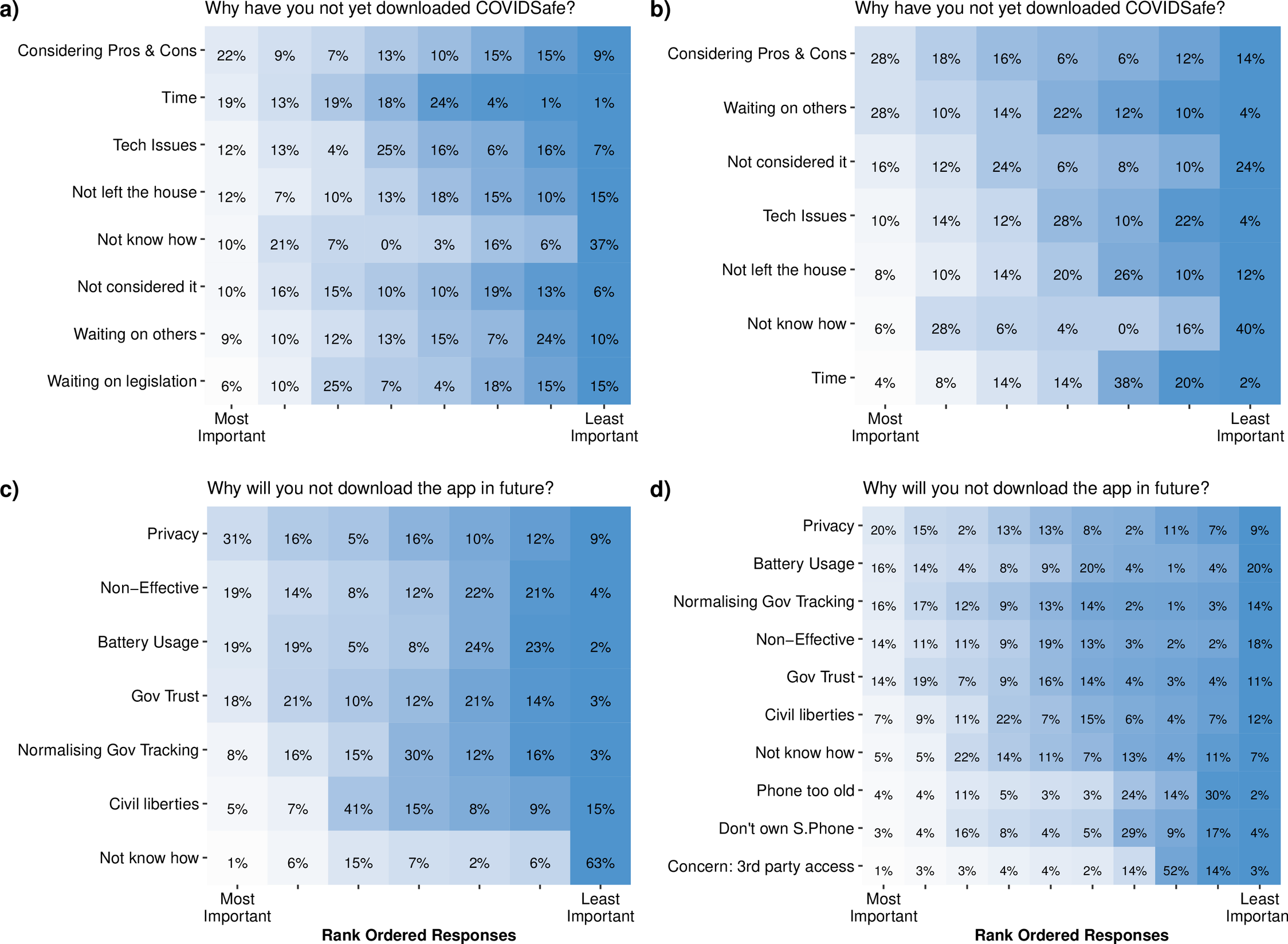

Supplement: S3 Fig — Rank ordered responses asking individuals why they are yet to download the COVIDSafe app in Samples 3 (a) and 4 (b), or why they will never download the app in Samples 3 (c) and 4 (d). Response percentages are displayed in text and cumulative responses probabilities for each item are displayed in blue. ‘Weighing the pros and cons’ was the primary reason for not yet downloading the app. In Sample 3 when the app was newly released, time, technical issues and not leaving the house were among the top reasons, however, were replaced in Sample 4 with less time-sensitive issues: ‘waiting on others’ and ‘not considered it’. In both samples, concerns regarding privacy, battery usage, Government trust, and a belief that the app will be non effective were primary reasons for never downloading COVIDSafe. In Sample 4, concern regarding the normalizing of Government tracking became a primary issue. (TIF) [file pone.0244827.s003.tif]
